# Evaluation of the PA-100 AST system (Sysmex) for point-of-care analysis of bacteriuria and antibiotic susceptibility testing in dogs and cats

**DOI:** 10.1128/spectrum.00811-26

**Published:** 2026-07-14

**Authors:** Sina Selent, Roswitha Dorsch, Georg Wolf, Karin Weber

**Affiliations:** 1 Small Animal Clinic, Centre for Clinical Veterinary Medicine, LMU Munich 584255, Munich, Germany; 2 Department of Bacteriology, Faculty of Veterinary Medicine, Institute of Infectious Diseases and Zoonoses, LMU Munich 267059, Munich, Germany; Michigan State University, East Lansing, Michigan, USA

**Keywords:** point-of-care, diagnostic, feline, canine, antibiotic stewardship, nanofluidics

## Abstract

Bacterial presence in urine (bacteriuria) is frequently observed in dogs and cats and may be associated with urinary tract infection (UTI), depending on the clinical context. However, no in-house test for the rapid detection of bacteriuria and subsequent antibiotic susceptibility tests (AST) is yet available. The Sysmex PA-100 AST System (PA-100) is a point-of-care (POC) analyzer using nanofluidic cartridges designed for the detection of bacteria and performance of AST directly from voided urine in female patients with cystitis symptoms. In this study, we tested the suitability of the PA-100 to detect UTIs and perform AST on veterinary samples. In total, 363 urine samples from dogs (*n* = 163) and cats (*n* = 200) collected via cystocentesis, catheter, endoscopy, or punctation of a ureteral bypass system were analyzed. The results were compared to routine microbiologic culture and AST. Using the manufacturer’s thresholds for bacteriuria, we observed a sensitivity of 87.1% (95% confidence interval [CI] 70.2–96.4), specificity of 81.7% (95% CI 73.6–88.1), positive predictive value (PPV) of 55.1% (CI 40.2–69.3), and negative predictive value (NPV) of 96.1% (95% CI 90.3–99.0) for UTIs of cats. For dogs, we observed a sensitivity of 87.1% (95% CI 70.2–96.4), specificity of 87.7% (95% CI 78.5–93.9), PPV of 73.0% (95% CI 55.9–86.2), and NPV of 94.7% (95% CI 86.9–98.5). The AST results of the PA-100 were concordant with the AST on plates in 90% of samples. The device has potential as a POC analyzer in the veterinary field, but a high proportion of false-positive bacteriuria results occurred. About 12% of samples yielded no results due to technical errors. Adjustments to the antibiotics used in the cartridges are required for veterinary medicine.

**IMPORTANCE:**

Currently available point-of-care (POC) tests for the detection of bacteriuria in cats and dogs show several limitations, and antimicrobial susceptibility testing (AST) is not provided by most of these tests. These POC tests generally necessitate incubation for more than 16 h. The Sysmex PA-100 AST System (PA-100), which is designed as a POC device to detect bacteriuria and perform AST-testing within 45 min in urine samples of female patients suffering from cystitis, was recently tested for the first time in a clinical setting in human medicine. Since timely results to detect bacteriuria and to guide antibiotic use are just as important in companion animals as they are in humans, this study aimed to evaluate the applicability of the PA-100 for veterinary use, testing urine samples from dogs and cats with clinical signs of cystitis. This technology may support faster urinalysis in veterinary patients. However, its current applicability in veterinary diagnostics is limited.

## INTRODUCTION

Urinary tract infections (UTIs) are one of the most common bacterial infections, both in humans and in companion animals, affecting millions of individuals each year and representing one of the leading causes for antibiotic prescriptions (1–4). The standard diagnostic approach relies on urine culture, which is considered the gold standard, but requires up to 72 h to deliver reliable results. During this diagnostic delay, empirical antibiotic therapy is frequently initiated in humans and dogs, which may result in inappropriate treatment and contribute to the global rise of antimicrobial resistance. Since most cats suffer from sterile cystitis, antibiotic treatment is typically postponed until the results of bacterial culture and antimicrobial susceptibility testing (AST) become available, which may unnecessarily prolong the disease in cases with UTI (3, 5). Given the clinical and epidemiological importance of UTIs and the increasing demand for strategies that support rational antibiotic use, the development and validation of reliable point-of-care (POC) diagnostics are of high relevance in human and in veterinary medicine. Several POC tests have previously been evaluated for the detection of bacteriuria in animals; however, most of these tests do not provide information on antimicrobial susceptibility, limiting their utility for guiding targeted therapy. By enabling both rapid detection and timely AST, such approaches have the potential to improve patient outcomes, decrease healthcare costs, and contribute to the containment of antimicrobial resistance within a One Health framework. In 2024, the first clinical evaluation of the Sysmex PA-100 AST (PA-100), a POC device for detecting bacteriuria and performing an antibiotic susceptibility test in human urine samples within 45 min, was published (6). The PA-100 identifies bacteriuria within 15 min and conducts phenotypic AST within 30 additional min by monitoring bacterial growth in Mueller-Hinton broth included in a nanofluidic cartridge with antibiotic-coated channels. The aim of this study was to evaluate the performance of the PA-100 to detect bacteriuria and perform AST in urine samples of dogs and cats with clinical signs of cystitis. To the author’s knowledge, this represents the first assessment of this technology in a veterinary context. It should be noted that the PA-100 is not validated for urine samples from dogs and cats, and speciation results should be interpreted with caution.

## MATERIALS AND METHODS

### Study design

A total of 200 urine samples from cats and 163 samples from dogs were included in this prospective study. These samples were submitted to the laboratory of the Small Animal Clinic at the Ludwig Maximilians University of Munich (LMU, Munich, Germany) for standard urinalysis by refractometry, dipstick, and sediment analysis. According to the instructions for use (IFU) of the PA-100, all urine samples were processed within the PA-100 within 30 min of collection, without prior refrigeration. Following PA-100 testing, samples were stored at 4°C–8°C and forwarded to the Institute of Infectious Diseases and Zoonoses, Department of Bacteriology, at the Faculty of Veterinary Medicine, LMU, Munich, for conventional bacteriological culture and antibiotic susceptibility testing. Sample handling and storage followed routine diagnostic laboratory procedures. Urine samples from client-owned dogs and cats were prospectively included when a bacteriological urine examination was clinically indicated. Eligible urine sample types comprised samples collected via cystocentesis, catheterization, endoscopy, or by puncture of a subcutaneous port of a ureteral bypass system. Samples from retests of the same patient were included only if collected and processed on different days. Animals that had received antimicrobial treatment were also included. Samples were excluded if they consisted of spontaneously voided urine or if more than 30 min had elapsed between urine collection and sample processing. A residual urine volume of 400 µL was required for analysis with the PA-100. The collection and use of urine samples for this study were approved by the ethics committee of the veterinary faculty of LMU Munich (approval no. 411-16-08-2024). The sample collection period extended from August 2024 to June 2025. The results obtained with the PA-100 had no impact on the antibiotic therapy of the animals, since the device is not approved for veterinary use.

#### Sample size calculation

An a priori sample size calculation for an exact McNemar test (paired proportions, two-sided) indicated that 144 paired samples were required, assuming α = 0.05, 80% power, an odds ratio of 2.0 (moderate effect size), and 50% discordant pairs (conservative assumption). The final number of included samples exceeded this minimum, further increasing statistical power and supporting the robustness of the results.

#### Routine analysis

Following sample collection, a standard urine analysis was performed. This included physical examination (color and turbidity), urine specific gravity measured by refractometry (RUR5-ATC refractometer), chemical analysis using a dipstick (IDEXX UA Strips and IDEXX VetLab UA analyzer), and automated urine sediment analysis (IDEXX SediVue Dx Urine Sediment Analyzer, IDEXX Laboratories Inc., Westbrook, ME, USA).

#### Manual microscopy

Each urine sample was examined briefly under the microscope. A drop of uncentrifuged urine was placed on a microscope slide, coverslipped, and examined at 100× magnification. Particular attention was paid to suspended particles, crystals, lipid droplets, and bacteria.

#### PA-100

A total of 400 µL of the collected fresh native urine sample was pipetted into a PA-100 cartridge. The workflow automatically starts after inserting the cartridge into the instrument. During the analysis, the urine sample first passes through a filter into a nanofluidic chip system. If bacterial cells are present, they become confined within individual channels, while larger components such as eukaryotic cells and other urinary particles are retained by the filter and do not enter the chip. In the following step, Mueller-Hinton broth II is directed through reservoirs containing dried antibiotics. As the broth passes through, it acquires a defined concentration of antibiotics and then flows into the channels where the bacteria are captured. All required reagents are preloaded into the cartridge. Once loading is complete, incubation begins, and a constant flow is maintained throughout the entire analysis. The nanofluidic chip contains approximately 11,000 nanochannels arranged into 32 sections, each containing several hundred individual channels. Distinct antibiotics and concentrations were applied to each section. Resistance profiles were generated by a proprietary algorithm calibrated against clinical breakpoint tables from the European Committee on Antimicrobial Susceptibility Testing (EUCAST) (7). During the analysis, a phase-contrast microscope captures images of all 32 sections at approximately 1 min intervals. A proprietary image-analysis algorithm compiled the image sequences into videos and calculated growth rates for each section, which were subsequently compared with a no-antibiotic control section. The system detected bacteriuria ≥50,000 CFU/mL (colony-forming units per milliliter) and reported AST outcomes as S (susceptible), I (susceptible, increased exposure), R (resistant), or not applicable for amoxicillin/clavulanic acid, ciprofloxacin, fosfomycin, nitrofurantoin, and trimethoprim, according to EUCAST guidelines (7). Bacteriuria was reported without disclosure of the presumptive bacterial species used to generate the AST result. The system provided the user with a binary output (bacteriuria positive/negative). Additional outcomes included E (technical error), LG (low growth; insufficient bacterial growth for reliable rapid AST determination), and NA (antimicrobial susceptibility testing was not applicable for the identified species, as per EUCAST recommendations). The cartridges were optimized for the detection of the most common uropathogens causing uncomplicated UTI in humans, namely *Escherichia coli*, *Klebsiella pneumoniae*, *Proteus mirabilis*, *Enterococcus faecalis*, and *Staphylococcus saprophyticus*. A proprietary, non-disclosed algorithm classified the detected bacteria into three groups (*Enterobacterales*, *Enterococcus*, and *Staphylococcus*) and applied the corresponding EUCAST breakpoints. Other species not within the IFU (manufacturer’s instructions for use) could also be detected and reported as positive; however, as the algorithm was not trained on these organisms, misclassification and inappropriate breakpoint assignment could occur, particularly for isolates with resistance values close to the breakpoint. For *S. saprophyticus* and *Enterococcus*, no EUCAST breakpoint for oral fosfomycin (human use) is available. In such cases, the analyzer reported “NA” (not applicable) for fosfomycin AST results. A quality control of the device was done every 4 weeks using PA-100 Control Beads in a standard cartridge. A detailed description of the underlying technology can be found elsewhere (8–12). According to the IFU, urine samples from patients who have been treated with antibiotics in the past 7 days should not be used. Also, samples with a high proportion of erythrocytes (macrohematuria) should not be analyzed with the PA-100, as these are associated with a higher error rate. The color of a urine sample provides an indication of when a sample is not recommended for analysis with the PA-100 AST system. According to the IFU, pale yellow to dark yellow samples show a lower risk of error, whereas light red to dark red samples have a higher risk of error (13). In this study, neither antibiotic pretreatment of the patient nor macrohematuria was an exclusion criterion.

#### Quantitative urine culture

Urine culture was performed at the Institute for Infectious Diseases and Zoonoses, Ludwig-Maximilians-University of Munich. Quantitative aerobic culture was carried out using Columbia agar with 5% sheep blood, Mueller-Hinton agar without sheep blood, and Gassner agar. All plates were inoculated with 0.1 mL of a 10^3^ dilution of the urine. The Columbia agar was additionally inoculated with 0.1 mL of undiluted urine. The plates were incubated aerobically at 37°C–38°C and examined daily for 3 days. The results were recorded in CFU per milliliter. Identification of every colony type was performed using a matrix-assisted laser desorption/ionization time of flight mass spectrometer (Sirius and MALDI Biotyper Identification-Software 3.1, Bruker Daltonics GmbH, Bremen, Germany).

#### Antimicrobial susceptibility testing

The antimicrobial susceptibility pattern was determined using the Kirby-Bauer disk diffusion method. Mueller-Hinton agar (Meck, Darmstadt, Germany) was inoculated with a visually (McFarland) defined quantity of bacteria, and antibiotic-impregnated discs (Oxoid Deutschland GmbH, Wesel, Germany) were placed on the surface. The standard panel of antimicrobials included 16 agents: doxycycline, trimethoprim-sulfadiazine, ampicillin, amoxicillin/clavulanic acid, cephalothin, cefovecin, nitrofurantoin, pradofloxacin, enrofloxacin, marbofloxacin, gentamicin, streptomycin, clindamycin, erythromycin, chloramphenicol, and colistin. The bacterial isolates were considered to be S, I, or R based on standardized zones of inhibition according to valid recommendations provided by the Clinical and Laboratory Standards Institute (CLSI; M31-A3 and Vet01-A4). These breakpoints were cross-checked against the latest veterinary CLSI guidelines (Vet01S, 7th Edition, 2024) to ensure consistency with current standards. For comparison with the PA-100 AST, additional plates were prepared with discs containing fosfomycin (200 µg), ciprofloxacin (5 µg), trimethoprim (5 µg), ampicillin (10 µg), and nitrofurantoin (100 µg). The inhibition zones of these substances were given in millimeters, measured using a Scan 4000 inhibition zone reader (INTERSCIENCE, 78860 Saint Nom la Bretêche—FRANCE) and interpreted using EUCAST criteria. This approach was chosen to align breakpoint interpretation for these antimicrobials with the EUCAST-based interpretation applied by the PA-100. As some EUCAST standards were missing for several isolates, the *E. coli* standard was applied in every case. The following standards were used for each antibiotic: fosfomycin (*Enterobacterales*, *E. coli* only [S ≥ 24 mm, R < 24 mm]), trimethoprim (*Enterobacterales* [S ≥ 15 mm, R < 15 mm]; *Staphylococcus* spp. [S ≥ 14 mm, R < 14 mm]), and ciprofloxacin (*Enterobacterales* [S ≥ 25 mm, R < 22 mm]; *Pseudomonas* spp. [S ≥ 50 mm, R < 26 mm]; *Acinetobacter* spp. [S ≥ 50 mm, R < 21 mm]; *Staphylococcus* spp., coagulase negative [S ≥ 50 mm, R < 22 mm]; *Enterococcus* spp. [S ≥ 15 mm, R < 15 mm]; *Pasteurella* spp. [S ≥ 27 mm, R < 27 mm]). It should be noted that the interpretation of susceptibility was based on these criteria, which were applied consistently across all isolates to ensure comparability of results.

### Statistical analysis

Data collection and statistical analysis were performed using Microsoft Excel (version 16.95.3) and Prism (version 5.04; GraphPad). The sensitivity and specificity of the PA-100 were calculated in comparison with microbiological culture using contingency tables with a 95% confidence interval (CI).

## RESULTS

### Study population and patient/sample characteristics

In total, 200 feline urine samples and 163 canine samples were collected in this study. Patient characteristics and collection methods are summarized in Table 1. The 200 feline samples were drawn from 175 individual cats; 157 cats (78.5%) were sampled once, 12 cats were sampled twice, 5 cats were sampled three times, and 1 cat was sampled four times. The 163 canine samples were drawn from 134 individual dogs; 110 dogs (67.5%) were sampled once, 20 dogs were sampled twice, 3 dogs were sampled three times, and one dog was sampled four times. A total of 40 urine samples from cats (20%) and 50 urine samples from dogs (30.7%) with prior antibiotic treatment within the last 4 weeks were enrolled (Table 2).

**TABLE 1:** Patient demographics and urine sample collection methods in cats (*n* = 175) and dogs (*n* = 134)^*a*^

		Cats	Dogs
Sex	Female	67 (38%)	73 (54%)
	Male	108 (62%)	61 (46%)
Age range and median (years)		0.3–20.3 (7.1)	0.3–16.3 (7.6)
Collection method	Cystocentesis	163 (81.5%)	140 (85.9%)
	Catheterization	26 (13%)	15 (9.2%)
	SP	11 (5.5%)	2 (1.2%)
	Endoscopy	0	6 (3.7%)

^*a*^ Some patients were sampled more than once (see Results). SP, puncture of a subcutaneous shunting port of a ureteral bypass system.

**TABLE 2:** Microbiological culture results in samples from cats and dogs with and without prior antibiotic treatment^*a*^

	<5 × 10^4^ CFU/mL growth in microbiological culture	≥5 × 10^4^ CFU/mL growth in microbiological culture	Total
Samples from cats with prior antibiotic treatment	32	8	40 (20%)
Samples from cats without prior antibiotic treatment	126	34	160 (80%)
Samples from dogs with prior treatment	38	12	50 (30.7%)
Samples from dogs without prior treatment	74	39	113 (69.3%)

^*a*^ A threshold of 5 × 10^4^ CFU/mL was applied in accordance with the PA-100 instructions for use (IFU) for the detection of bacteriuria. All types of isolates are represented in this table, including those not covered by the IFU.

### Detection of bacteriuria

The performance of the PA-100 to detect bacteriuria was compared to the standard microbiological culture as the reference. In total, 42 feline urine samples with >5 × 10^4^ CFU/mL were identified by microbiological culture, and 51 canine urine samples were identified (21% and 31% of all samples, respectively). Among these, 35 monoinfections were observed in cat samples and 43 in dog samples; the remaining samples showed mixed infections (Table 3). The isolates detected by microbiological culture and the number of samples in which bacteriuria was detected with the PA-100 are shown in Table 4 (feline samples) and Table 5 (canine samples). As expected, the most common isolate was *E. coli*. Isolates within the IFU made up 73% of bacteriuria-positive samples. The overall detection rate for bacteriuria of the PA-100 was 78.6% for feline samples and 70.6% for canine samples.

**TABLE 3:** Results for bacteriuria ≥5 × 10^4^ CFU/mL by standard microbiological culture for at least one bacterial species^*a*^

	Bacteriuria ≥5 × 10^4^ CFU/mL	Monoinfections ≥5 × 10^4^ CFU/mL	Mixed infections: first and second isolate ≥5 × 10^4^ CFU/mL
Cat samples	42	35	7
Dog samples	51	43	8

^*a*^ All types of isolates are represented in this table, including those not covered by the PA-100 IFU.

**TABLE 4:** Summary of microbiological culture results and detection of bacteriuria by the PA-100 in cats^*a*^

	Microbiological culture	Bacterial isolates ≥5 × 10^4^ CFU/mL	Detected by PA-100 (bacteriuria positive)
Gram negative	***Escherichia coli***	23	22
	*Acinetobacter baumannii*	2	2
	*Pasteurella multocida*	2	1
	*Acinetobacter lwoffii*	1	1
	***Klebsiella pneumoniae***	1	1
	*Pasteurella dagmatis*	1	1
	***Proteus mirabilis***	1	1
Subtotal		31	29
Gram positive	***Enterococcus faecalis***	7	3
	*Staphylococcus felis*	3	0
	*Staphylococcus pseudintermedius*	1	1
Subtotal		11	4
	Total	42	33 (78.6%)

^*a*^ Bacterial species in bold are within the specification of the PA-100. A threshold of 5 × 10^4^ CFU/mL in urine was applied in accordance with the PA-100 instructions for use (IFU) for the detection of bacteriuria.

**TABLE 5:** Summary of microbiological culture results and detection of bacteriuria by the PA-100 in dogs^*a*^

	Microbiological culture	Bacterial isolates ≥5 × 10^4^ CFU/mL	Detected by PA-100 (bacteriuria positive)
Gram negative	***Escherichia coli***	28	22
	***Proteus mirabilis***	5	2
	*Acinetobacter baumannii*	1	1
	***Klebsiella pneumoniae***	1	1
	*Pseudomonas aeruginosa*	1	1
	*Raoultella ornithinolytica*	1	1
Subtotal		37	28
Gram positive	*Staphylococcus pseudintermedius*	6	5
	*Enterococcus faecium*	3	1
	***Enterococcus faecalis***	2	1
	*Corynebacterium urealyticum*	1	0
	*Enterococcus raffinosus*	1	0
Subtotal		13	7
Other	*Mycoplasma*	1	1
Total		51	36 (70.6%)

^*a*^ Bacterial species in bold are within the specification of the PA-100. A threshold of 5 × 10^4^ CFU/mL in urine was applied in accordance with the PA-100 instructions for use (IFU) for the detection of bacteriuria.

The detection of mixed infections has not been evaluated with the PA-AST panels. Performance for species other than *E. coli*, *K. pneumoniae*, *P. mirabilis*, *S. saprophyticus*, and *E. faecalis* has not been evaluated yet. Other uropathogens within the intended patient population may lead to a false result for bacteriuria or a false AST result. Species that do not grow in the cartridges will lead to a negative bacteriuria result (13). The study demonstrates that the device was able to detect pathogens not included in its specifications (Tables 4 and 5) and, in some cases, even generated an antibiogram. In 27 feline and 15 canine samples, the PA-100 reported bacteriuria, but did not display a result for antimicrobial susceptibility testing (E(5) error). More than 60% of these samples were negative by microbiological culture (Table 6).

**TABLE 6:** Samples with a PA-100 bacteriuria positive result displaying an E (5) error event (no antimicrobial susceptibility testing [AST] result displayed)^*a*^

Microbiological culture result	Bacterial concentration in CFU/mL	E (5) error in feline samples	E (5) error in canine samples
Bacterial isolates within the IFU	≥5 × 10^4^	5	2
	<5 × 10^4^	0	1
Bacterial isolates outside the IFU	≥5 × 10^4^	2	1
	<5 × 10^4^	2	1
Negative (no bacterial growth)	0	18	10
Total		27	15

^*a*^ Results below and above the bacterial concentration threshold (5 × 10^4^ CFU/mL) according to the IFU of the PA-100 and with bacterial isolates within or outside the specifications (IFU) of the device are included. Note the high number of samples with a positive PA-100 bacteriuria result, but a negative result in microbiological culture.

The performance characteristics for the detection of bacteriuria of the PA-100 are shown in Table 7 (feline samples) and Table 8 (canine samples). The calculations for sensitivity, specificity, positive predictive value (PPV), and negative predictive value (NPV) were done using different cut-offs for the bacterial concentration: a calculation with no cut-off including all samples with a positive result in microbiological culture, a calculation for a cut-off of ≥1 × 10^3^ CFU/mL, and a calculation for a cut-off of ≥5 × 10^4^ CFU/mL. Additional calculations were done to include only samples with bacterial isolates within the IFU of the PA-100. The cut-off ≥1 × 10^3^ CFU/mL represents the threshold for clinically relevant bacteriuria in feline and canine urine samples gained by cystocentesis, and the cut-off ≥5 × 10^4^ CFU/mL represents the threshold of the PA-100 detection limit as specified in the manual. The tables also include the numbers for samples that did not produce a valid result in the PA-100. For feline urine samples, the sensitivity ranges from 58.3% to 87.1%, the specificity is at 81.7%, the PPV ranges from 55.1% to 61.4%, and the NPV ranges from 79.7% to 96.1%. The PA-100 did not produce a valid result in about 10% of feline samples due to technical errors. For canine urine samples, the sensitivity ranges from 62.3% to 87.1%, the specificity ranges from 87.6% to 87.7%, the PPV ranges from 73.0% to 79.2%, and the NPV ranges from 75.5% and 94.7%. The PA-100 did not produce a valid result in about 13.5% of canine samples due to technical errors.

**TABLE 7:** Feline samples: sensitivity, specificity, positive, and negative predictive value for the detection of bacteriuria by the PA-100 compared to microbiological culture^*a*^^,^^*b*^

	Culture positive No cut-off value All bacterial isolates	Culture negative	Total
PA-100 positive	35	22	57
PA-100 negative	25	98	123
PA-100 no result	5	15	20
Total	65	135	200
Performance	Sensitivity (95% CI) 58.3% (44.9%–70.9%) Specificity (95% CI) 81.7% (73.6%–88.1%) PPV (95% CI) 61.4% (47.6%–74.0%) NPV (95% CI) 79.7% (71.5%–86.4%)		
“No result“ cases (*n* = 20) were excluded from performance calculations			

	Culture positive Cut-off value ≥ 1 × 10^3^ All bacterial isolates	Culture negative	Total
PA-100 positive	33	22	55
PA-100 negative	13	98	111
PA-100 no result	2	15	17
Total	48	135	183
Performance	Sensitivity (95% CI) 71.7% (56.5%–84.0%) Specificity (95% CI) 81.7% (73.6%–88.1%) PPV (95% CI) 60.0% (45.9%–73.0%) NPV (95% CI) 88.3% (80.8%–93.6%)		
“No result“ cases (*n* = 17) were excluded from performance calculations			

	Culture positive Cut-off value ≥ 1 × 10^3^ IFU bacterial isolates	Culture negative	Total
PA-100 positive	27	22	49
PA-100 negative	9	98	107
PA-100 no result	1	15	16
Total	37	135	172
Performance	Sensitivity (95% CI) 75.0% (57.8%–87.9%) Specificity (95% CI) 81.7% (73.6%–88.1%) PPV (95% CI) 55.1% (40.2%–69.3%) NPV (95% CI) 91.6% (84.6%–96.1%)		
“No result“ cases (*n* = 16) were excluded from performance calculations			

	Culture positive Cut-off value ≥ 5 × 10^4^ All bacterial isolates	Culture negative	Total
PA-100 positive	33	22	55
PA-100 negative	7	98	105
PA-100 no result	2	15	17
Total	42	135	177
Performance	Sensitivity (95% CI) 82.5% (67.2%–92.7%) Specificity (95% CI) 81.7% (73.6%–88.1%) PPV (95% CI) 60.0% (45.9%–73.0%) NPV (95% CI) 93.3% (86.6%–97.3%)		
“No result“ cases (*n* = 17) were excluded from performance calculations			

	Culture positive Cut-off value ≥ 5 × 10^4^ IFU bacterial isolates	Culture negative	Total
PA-100 positive	27	22	49
PA-100 negative	4	98	102
PA-100 no result	1	15	16
Total	32	135	167
Performance	Sensitivity (95% CI) 87.1% (70.2%–96.4%) Specificity (95% CI) 81.7% (73.6%–88.1%) PPV (95% CI) 55.1% (40.2%–69.3%) NPV (95% CI) 96.1% (90.3%–99.0%)		
“No result“ cases (*n* = 16) were excluded from performance calculations			

^*a*^ The results are displayed for different cut-offs for bacterial concentration (no cut-off; cut-off ≥ 1 × 10^3^ CFU/mL; cut-off ≥ 5 × 10^4^ CFU/mL) and for including all bacterial isolates detected by microbiological culture or including only bacterial isolates covered by the instructions for use (IFU) of the PA-100 (*E. coli*,* P. mirabilis*,* K. pneumoniae*,* S. saprophyticus*, and* E. faecalis*).

^*b*^ CI, confidence interval; PPV, positive predictive value; NPV, negative predictive value.

**TABLE 8:** Canine samples: sensitivity, specificity, positive and negative predictive value for the detection of bacteriuria by the PA-100 compared to microbiological culture^*a*^^,^^*b*^

	Culture positive No cut-off value All bacterial isolates	Culture negative	Total
PA-100 positive	38	10	48
PA-100 negative	23	71	94
PA-100 no result	8	13	21
Total	69	94	163
Performance	Sensitivity (95% CI) 62.3% (49.0%–74.4%) Specificity (95% CI) 87.6% (78.5%–93.9%) PPV (95% CI) 79.2% (65.0%–89.5%) NPV (95% CI) 75.5% (65.6%–83.8%)		
“No result” cases (*n* = 21) were excluded from performance calculations			

	Culture positive Cut-off value ≥ 1 × 10^3^ All bacterial isolates	Culture negative	Total
PA-100 positive	37	10	47
PA-100 negative	11	71	82
PA-100 no result	8	13	21
Total	56	94	150
Performance	Sensitivity (95% CI) 77.1% (62.7%–88.0%) Specificity (95% CI) 87.7% (78.5%–93.9%) PPV (95% CI) 78.7% (64.3%–89.3%) NPV (95% CI) 86.6% (77.3%–93.1%)		
“No result” cases (*n* = 21) were excluded from performance calculations			

	Culture positive Cut-off value ≥ 1 × 10^3^ IFU bacterial isolates	Culture negative	Total
PA-100 positive	28	10	38
PA-100 negative	6	71	77
PA-100 no result	5	13	18
Total	39	94	133
Performance	Sensitivity (95% CI) 82.4% (65.5%–93.2%) Specificity (95% CI) 87.7% (78.5%–93.9%) PPV (95% CI) 73.7% (56.9%–86.6%) NPV (95% CI) 92.2% (83.8%–97.1%)		
“No result” cases (*n* = 18) were excluded from performance calculations			

	Culture positive Cut-off value ≥ 5 × 10^4^ All bacterial isolates	Culture negative	Total
PA-100 positive	36	10	46
PA-100 negative	9	71	80
PA-100 no result	6	13	19
Total	51	94	145
Performance	Sensitivity (95% CI) 80.0% (65.4%–90.4%) Specificity (95% CI) 87.7% (78.5%–93.9%) PPV (95% CI) 78.3% (63.6%–89.1%) NPV (95% CI) 88.8% (79.7%–94.7%)		
“No result” cases (*n* = 19) were excluded from performance calculations			

	Culture positive Cut-off value ≥ 5 × 10^4^ IFU bacterial isolates	Culture negative	Total
PA-100 positive	27	10	37
PA-100 negative	4	71	75
PA-100 no result	5	13	18
Total	36	94	130
Performance	Sensitivity (95% CI) 87.1% (70.2%–96.4%) Specificity (95% CI) 87.7% (78.5%–93.9%) PPV (95% CI) 73.0% (55.9%–86.2%) NPV (95% CI) 94.7% (86.9%–98.5%)		
“No result” cases (*n* = 18) were excluded from performance calculations			

^*a*^ The results are displayed for different cut-offs for bacterial concentration (no cut-off; cut-off ≥1 × 10^3^ CFU/mL; cut-off ≥5 × 10^4^ CFU/mL) and for including all bacterial isolates detected by microbiological culture or including only bacterial isolates covered by the instructions for use (IFU) of the PA-100 (*E. coli*,* P. mirabilis*,* K. pneumoniae*,* S. saprophyticus*, and* E. faecalis*).

^*b*^ CI, confidence interval; PPV, positive predictive value; NPV, negative predictive value; IFU, instructions for use.

### Performance of PA-100 AST results

The analytical performance of the PA-100 was evaluated by comparing its antibiograms with those of the microbiologic reference laboratory. Only samples with a bacteriuria ≥5 × 10^4^ CFU/mL and with bacteria isolates within the IFU of the PA-100 were included. Due to multiple error messages, only a fraction of these samples could be evaluated. While most results of the PA-100 were in concordance with the results of microbiological culture, about 10% were assigned in the opposite group (Culture susceptible vs PA-100 resistant and vice versa; Tables 9 and 10).

**TABLE 9:** Feline samples, performance of PA-100 for antimicrobial susceptibility testing (AST)^*a*^

Microbiological reference	PA-100	AMC	CIP	FOS	NIT	TRI
**S**	S	17 (85%)	17 (85%)	15 (94%)	18 (86%)	15 (83%)
	I	2 (10%)	3 (15%)		1 (5%)	
	R	1 (5%)		1 (6%)	2 (9%)	3 (17%)
	total	20	20	16	21	18
**I**	S				1 (100%)	
	I					
	R		1 (100%)			
	total		1		1	
**R**	S	1 (50%)		2 (67%)		
	I					
	R	1 (50%)		1 (33%)		1 (100%)
	total	2		3		1
Frequency of no result due to NA				2		2
Frequency of no result due to E (5)			1			
Frequency of no result due to LG				1		1

^*a*^ Only samples positive for bacterial isolates within the instructions for use (IFU) and above the cut-off ≥ 5 × 10^4^ CFU/mL were evaluated. PA-100 results were compared against reference methods: disc diffusion for amoxicillin/clavulanic acid (AMC), ciprofloxacin (CIP), fosfomycin (FOS), nitrofurantoin (NIT), and trimethoprim (TRI). S, susceptible; I, susceptible, increased exposure; R, resistant, based on standard dosing regimen. “NA” indicates that antimicrobial susceptibility testing was not applicable for the identified species, as per EUCAST recommendations. “LG” indicates a bacterial growth rate too low for accurate AST results. “E (5)” indicates a technical error, and no AST result could be generated.

**TABLE 10:** Canine samples, performance of PA-100 for antimicrobial susceptibility testing (AST)^*a*^

Microbiological reference	PA-100	AMC	CIP	FOS	NIT	TRI
**S**	S	21 (91%)	20 (95%)	17 (100%)	17 (85%)	17 (85%)
	I					
	R	2 (9%)	1 (5%)		3 (15%)	3 (15%)
	Total	23	21	17	20	20
**I**	S		1 (100%)		1 (100%)	
	I					
	R					
	Total		1		1	
**R**	S			4 (80%)		
	I					
	R		1 (100%)	1 (20%)		2 (100%)
	Total		1	5	0	2
Frequency of no result due to NA				1		1
Frequency of no result due to E (5)			1	1	1	1
Frequency of no result due to LG		1			2	

^*a*^ Only samples positive for bacterial isolates within the instructions for use (IFU) and above the cut-off ≥ 5 × 10^4^ CFU/mL were evaluated. PA-100 results were compared against reference methods: disc diffusion for amoxicillin/clavulanic acid (AMC), ciprofloxacin (CIP), fosfomycin (FOS), nitrofurantoin (NIT), and trimethoprim (TRI). S, susceptible; I, susceptible, increased exposure; R, resistant, based on standard dosing regimen. “NA” indicates that antimicrobial susceptibility testing was not applicable for the identified species, as per EUCAST recommendations. “LG” indicates a bacterial growth rate too low for accurate AST results. “E (5)” indicates a technical error, and no AST result could be generated.

### AST analysis restricted to *E. coli*

To improve the interpretability of AST comparisons, an additional analysis was performed, including only *E. coli* isolates, as this isolate represented the most frequently detected uropathogen in the study population. A total of 74 *E. coli* isolates were included in this analysis. The categorical agreement between the PA-100 and conventional AST was evaluated for all antimicrobial agents tested. The results of this comparison are summarized in Fig. S1 to S5 as supplemental material.

## DISCUSSION

To our knowledge, this study documents for the first time the performance of the PA-100 in the veterinary field using urine samples of cats and dogs. We decided to use the system in a veterinary clinical setting without excluding animals with antibiotic pretreatment or samples with microhematuria. The cut-offs of ≥1 × 10^3^ CFU/mL and ≥5 × 10^4^ CFU/mL were chosen to reflect the threshold for significant bacteriuria in cystocentesis samples from dogs and cats and the threshold for voided urine as specified in the IFU of the PA-100.

An ideal POC device for veterinary use should meet the cut-off of ≥1 × 10^3^ CFU/mL, since most samples are gained by cystocentesis (14–16). As expected, the sensitivity, PPV, and NPV of the PA-100 are lower with this cut-off than with the cut-off specified in the IFU.

The first clinical evaluation of the PA-100 in a near-patient setting in human medicine found a sensitivity of 84.0% (95% CI: 75.6%–90.4%) and a specificity of 99.4% (95% CI 96.5%–100%) for detecting microbiologically confirmed bacteriuria in samples with >5 × 10^4^ CFU/mL and with bacterial species covered by the analyzer’s IFU (6). Using comparable specifications, the analyzer reached a sensitivity of 87.1% (95% CI: 70.2%–96.4%) in feline and canine samples, a specificity of 81.7% (95% CI 73.6%–88.1%) in feline samples, and a specificity of 87.7% (95% CI 78.5%–93.9%) in canine samples. As shown in Tables 7 and 8, the PA-100 gives a high proportion of false positive results for bacteriuria in feline and canine samples. This may be caused by formed elements other than bacteria in the urine sediment, or by lipid droplets, which are much more common in the urine of dogs and cats than in human urine (17).

We analyzed all samples both with manual microscopy and an automated sediment analyzer but could not find any specific reason for false positive results. Based on our observations, lipid droplets and erythrocytes do not interfere with the PA-100 detection process. Furthermore, 10%–14% of samples did not give a valid result at all, and the instrument returned an error message. The error messages were not dependent on the clarity, color, or number of cells and crystals of the samples, since valid results could be gained in samples with high lipid content, macrohematuria, and pyuria, and error messages also occurred in samples with low lipid content and an unremarkable sediment. The reasons for the invalid measurements remain unknown and may not all be sample related. Due to cost restraints, we could not retest these samples with a new cartridge, as recommended by the instrument in case of failure.

A potential limitation of this study is the difference in sample handling between the PA-100 testing and conventional bacteriological culture. While urine samples were processed with the PA-100 within 30 min of collection in accordance with the IFU, samples were refrigerated prior to culture and AST. Although this workflow reflects routine clinical practice, differences in processing time and storage conditions may have influenced bacterial viability and growth and should be considered when interpreting comparative results.

An important limitation of this study relates to the use of different antimicrobial susceptibility breakpoints. The PA-100 uses EUCAST standards as the default reference for antimicrobial susceptibility testing. While this approach is well established in human clinical microbiology, there are limitations in the context of veterinary medicine. EUCAST breakpoints are defined exclusively for human pathogens and therapies (7). For data evaluation, EUCAST standards were applied to the interpretation of inhibition zones on the antibiotic plates that had been specifically prepared in the microbiologic laboratory (CIP, TRI, and FOS) to align with the interpretive framework used by the PA-100. When no EUCAST standard for bacterial species was available, we always applied the standard of *E. coli.* The conventional disk diffusion testing was primarily interpreted using CLSI veterinary breakpoints for dogs and cats, where available. Differences between EUCAST and CLSI veterinary breakpoints may have influenced susceptibility categorization and limited direct comparability between methods. To address this limitation, an additional analysis restricted to the *E. coli* isolates was performed, allowing interpretation based on the same EUCAST criteria and thereby improving comparability between methods.

In the present study, *E. coli* was the most common uropathogen detected in feline and canine urine samples, which agrees with several previous veterinary studies (18–22) and with the study of Alonso-Tarrés et al. in human urine samples (6). In human voided urine, 65 out of 74 samples (88%) with *E. coli* bacteriuria above the threshold of ≥5 × 10^4^ CFU/mL were detected by the PA-100, which is comparable to our study, where 44 out of 51 samples (86%) with *E. coli* bacteriuria were detected. *Klebsiella pneumoniae* isolates were very rare in canine and feline urine samples but made up about 16% of bacteriuria in human samples. In our samples, 3 out of 6 (50%) *Proteus mirabilis* isolates were detected by the PA-100. This isolate was rare in human samples, and 2 out of 3 were detected by the device (6). The detection rate of *Enterococcus faecalis* in the present study, with 4 out of 5 samples detected by the PA-100 in feline and canine samples, was also comparable to 2 out of 4 in human samples (6). Our study did not document any urine samples containing *Staphylococcus saprophyticus*. Instead, isolates of *Staphylococcus felis* and *pseudintermedius* were detected. While it can be assumed that these species exhibit growth behavior comparable to *Staphylococcus saprophyticus*, we did not include these samples as “within IFU.” Although we did not get as many valid detection results, the rate of positive samples within the IFU is comparable between our study and that of Alonso-Tarrés et al. (73% vs 71%) (6). All other isolates listed in Tables 4 and 5 were outside the IFU. Nevertheless, the device was able to detect most of these non-specific bacterial species, even though the IFU states that a negative bacteriuria result is usually expected with bacteriuria outside the specification (13). Alonso-Tarrés et al. also documented multiple isolates outside the IFU; some of them could also be detected by the PA-100 (6). It remains unclear how the PA-100 was able to detect species not included in the IFU, such as *Mycoplasma* spp. or *Acinetobacter baumanii*, whereas the slow-growing *Corynebacterium urealyticum* likely went undetected due to its growth characteristics. Notably, *Mycoplasma* species typically require specialized culture media for growth, which are not provided in standard testing conditions. Furthermore, only a single *Corynebacterium urealyticum* isolate exceeded the detection cut-off in this study, limiting the evaluation of the system’s performance for this species. These observations highlight a limitation of the current system, as certain slow-growing veterinary uropathogens may be missed. Expanding the bacterial spectrum to include such organisms would be important to allow more comprehensive application of the PA-100 in clinical practice.

According to the IFU, the PA-100 is only described as detecting monoinfections—it has not yet been tested for the detection of mixed infections. About 5% of our samples showed mixed infections. We assumed that the bacterium with the higher colony count was more dominant in the cartridge than the other isolates present in the sample and used this assumption for our calculations. However, if a mixed infection, in which one or more additional bacterial species proliferate to a clinically relevant extent, is not recognized, these co-pathogens may influence the selection of an appropriate antibiotic therapy.

Since the PA-100 did not produce AST results in all our culture-positive samples due to measurements with error messages, only a limited comparison to human urine samples is possible. Susceptible isolates were correctly identified by the PA-100 in human urine samples with a high accuracy (92%–96%), while the performance was slightly lower in our samples (83%–100%). Resistant isolates were found more often in human urine samples (about 20% over all antibiotics) than in our samples (about 7%); the number of resistant isolates in our study was too low for statistical evaluation. In the study of Alonso-Tarrés et al. (6), only the error message “NA” was recorded, while we also encountered the error codes “LG” and “E.” It is unclear whether these errors did not occur when analyzing human urine samples or whether they were not reported. In the present study, we tested each sample only once. Repeated testing in case of an error might have improved the number of valid results.

The antibiotic panels in the current PA-100 cartridge contain amoxicillin/clavulanic acid, ciprofloxacin, fosfomycin, nitrofurantoin, and trimethoprim. To implement the PA-100 in routine veterinary practice, panels specifically designed for veterinary use would be required. Of the antibiotics tested, only amoxicillin/clavulanic acid is relevant for veterinary application. Nitrofurantoin could also be used but is generally avoided because it should be only considered for sporadic cystitis where first-line antibiotics are not appropriate due to culture and AST (3). The remaining three antibiotics have no application in veterinary practice and only reflect the pre-set antimicrobial panel of the PA-100 cartridge and were used for technical evaluation only. Their use in dogs and cats is limited or contraindicated, and this study does not intend to provide therapeutic recommendations.

The economic aspects of implementing the PA-100 should be considered. The device has an acquisition cost of approximately €5,000, while each urine analysis requires a single-use cartridge costing approximately €35. External quality control materials incur an additional cost of approximately €70 per set. These figures provide a basis for evaluating the cost-effectiveness and practical feasibility of routine POC testing in a veterinary clinical setting.

In conclusion, the PA-100 demonstrates potential for the analysis of urine samples from dogs and cats, but its current applicability in veterinary diagnostics is limited. The bacterial spectrum is mostly appropriate for the clinical needs of veterinary medicine, but the lack of testing options for antimicrobials authorized for use in dogs and cats, such as amoxicillin without clavulanic acid, trimethoprim-sulfadiazine, marbofloxacin, and pradofloxacin, represents a considerable drawback. Even more concerning, we encountered a high number of error messages during testing. Without a systematic evaluation of whether these errors are device or sample related, the performance of the PA-100 for POC use in the veterinary field remains questionable. If these requirements are met, the PA-100 has the potential to serve as a user-friendly POC device that could significantly improve targeted antimicrobial treatment in dogs and cats with sporadic cystitis and help to avoid unnecessary antimicrobial use.

## SUPPLEMENTAL MATERIAL

Supplemental material spectrum.00811-26-s0001.pdf Distribution of inhibition zone diameters of *E. coli* isolates for nitrofurantoin, trimethoprim, amoxiclav, ciprofloxacin, and fosfomycin.

ASM does not own the copyrights to Supplemental Material that may be linked to, or accessed through, an article. The authors have granted ASM a non-exclusive, world-wide license to publish the Supplemental Material files. Please contact the corresponding author directly for reuse.
