# Supplementary material for: Evaluation of the PA-100 AST system (Sysmex) for point-of-care analysis of bacteriuria and antibiotic susceptibility testing in dogs and cats
Source: Microbiol Spectr. 2026 Jul 14;14(8):e00811-26. doi: 10.1128/spectrum.00811-26 (PMC13436350; doi:10.1128/spectrum.00811-26)

SUPPLEMENTARY MATERIAL

Figure 1 Distribution of inhibition zone diameters (AGID, in mm) of E. coli isolates against nitrofurantoin. Bar colors indicate phenotype: green = PA-100 and AGID “S” (susceptible), grey = PA-100 “R”; AGID “S” (resistant by PA-100, but susceptible by AGID), yellow = PA-100 “S”; AGID “I” (intermediate).

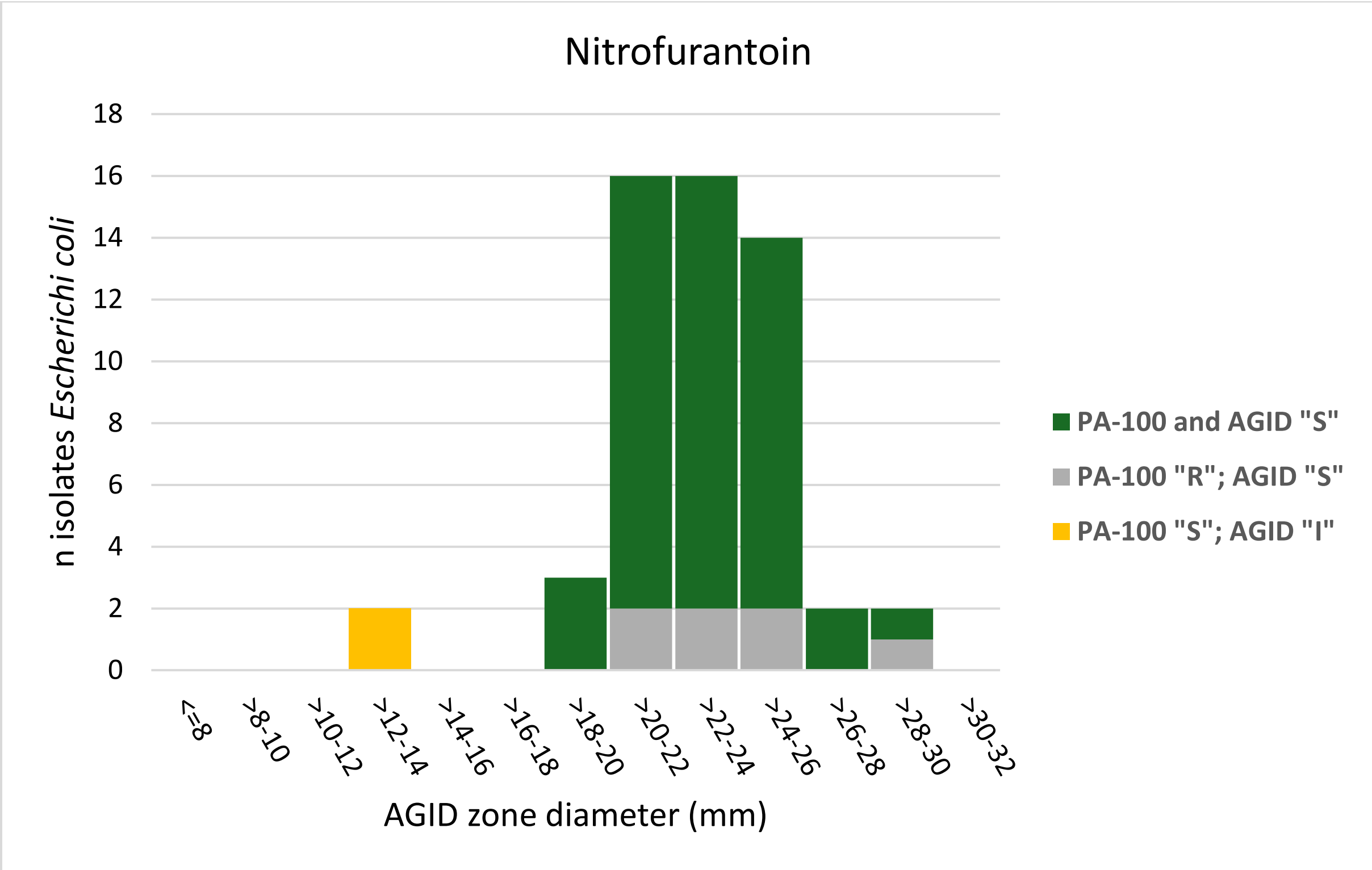

Figure 2 Distribution of inhibition zone diameters (AGID, in mm) of E. coli isolates against trimethoprim. Bar colors indicate phenotype: green = PA-100 and AGID “S“ (susceptible), blue = PA-100 and AGID “R“ (resistant), grey = PA-100 “R“; AGID “S“ (resistant by PA-100 but susceptible by AGID).

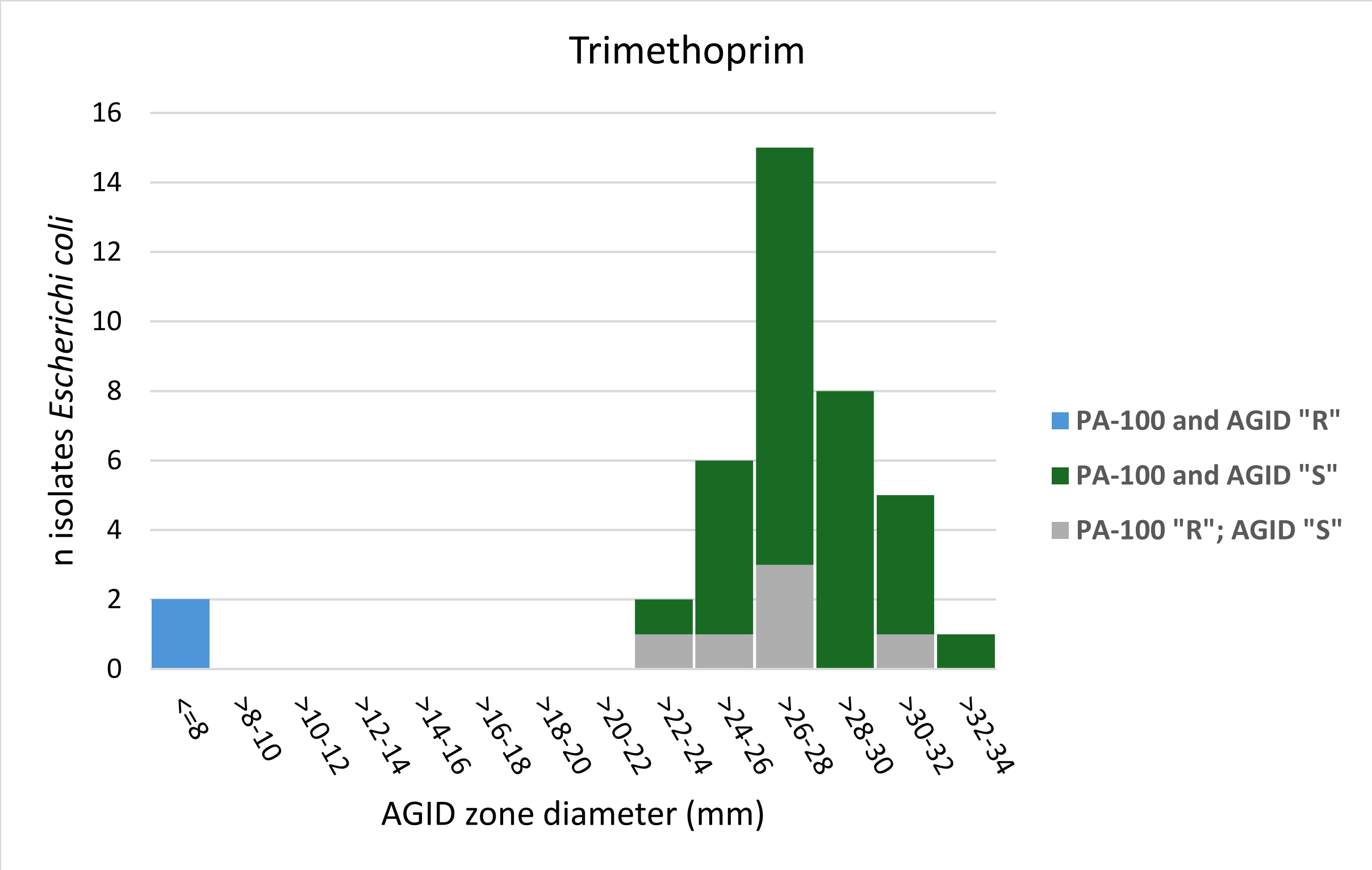

Figure 3 Distribution of inhibition zone diameters (AGID, in mm) of E. coli isolates against amoxicillin-clavulanic acid. Bar colors indicate phenotype: green = PA-100 and AGID “S“ (susceptible), blue = PA-100 and AGID “R” (resistant), orange = PA-100 “R”; AGID “I” (resistant by PA-100 but intermediate by AGID), grey = PA-100 “R”; AGID “S” (resistant by PA-100 but susceptible by AGID).

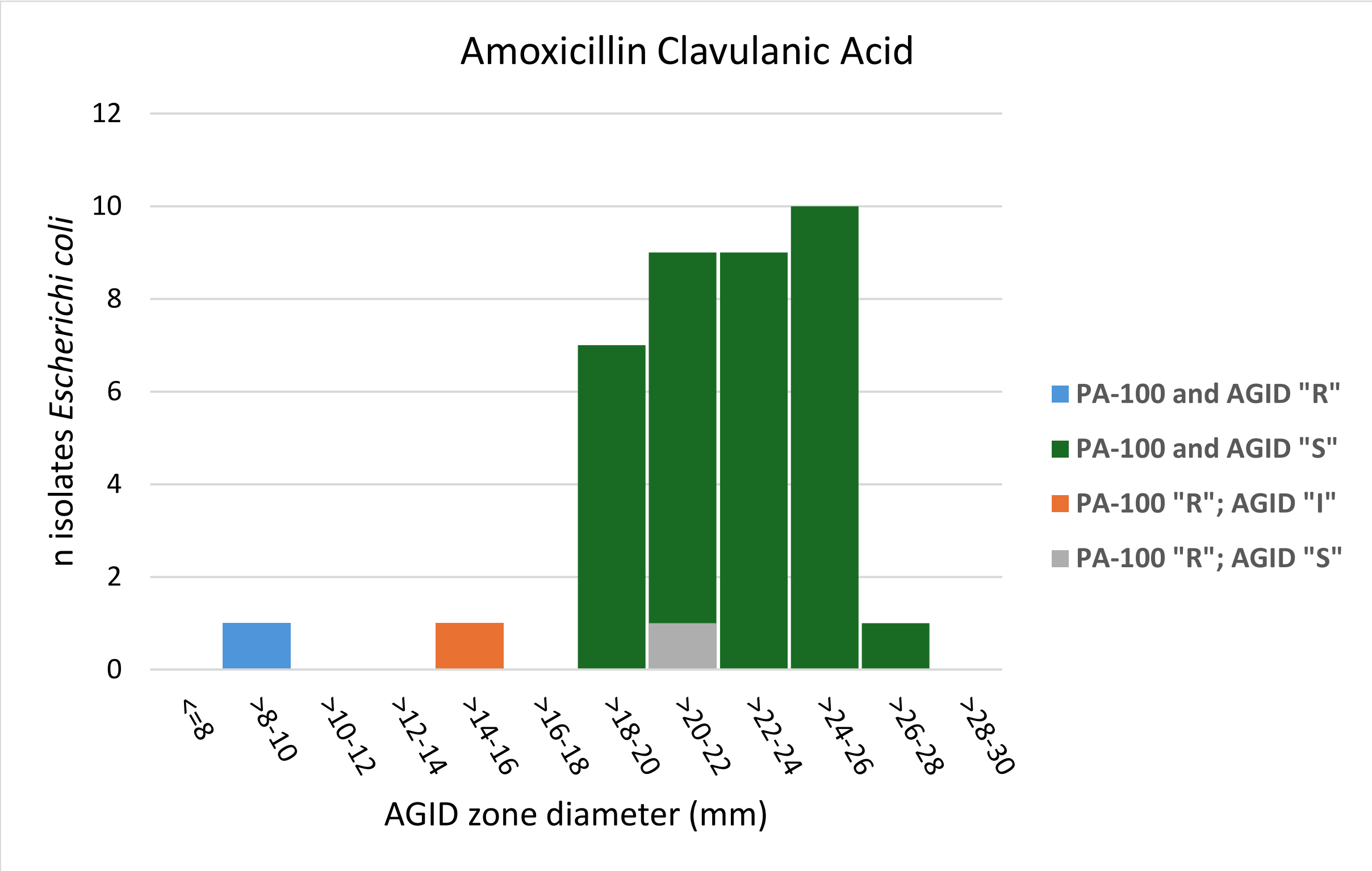

Figure 4 Distribution of inhibition zone diameters (AGID, in mm) of E. coli isolates against ciprofloxacin. Bar colors indicate phenotype: green = PA-100 and AGID “S“ (susceptible), pink = PA-100 “I“; AGID “S“ (intermediate by PA-100 but susceptible by AGID), grey = PA-100 “R“; AGID “S“ (resistant by PA-100 but susceptible by AGID).

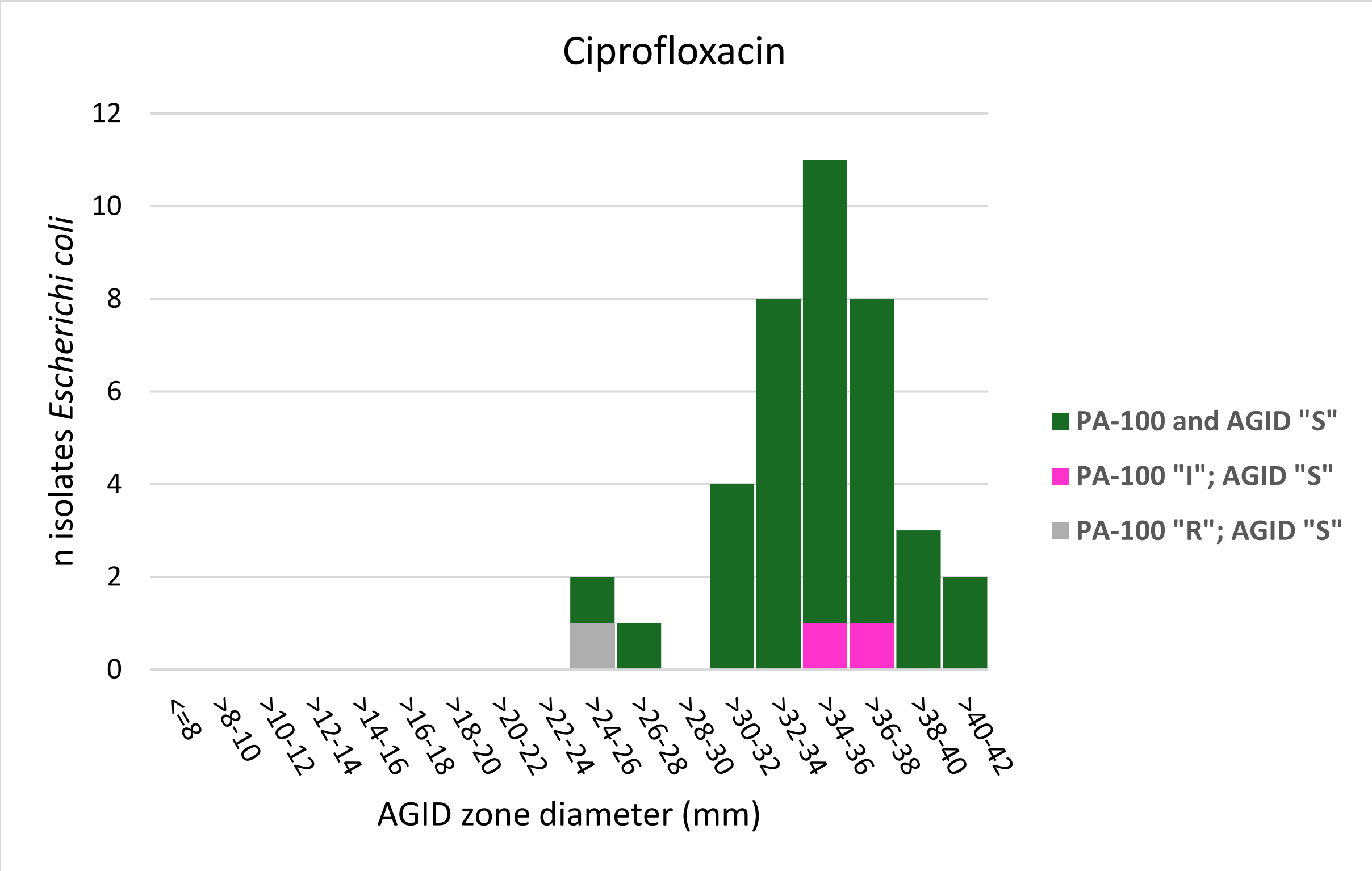

Figure 5 Distribution of inhibition zone diameters (AGID, in mm) of E. coli isolates against fosfomycin. Bar colors indicate phenotype: green = PA-100 and AGID “S” (susceptible), brown = PA-100 “S”; AGID “R” (susceptible by PA-100 but resistant by AGID), grey = PA-100 “R”; AGID “S” (resistant by PA-100 but susceptible by AGID).

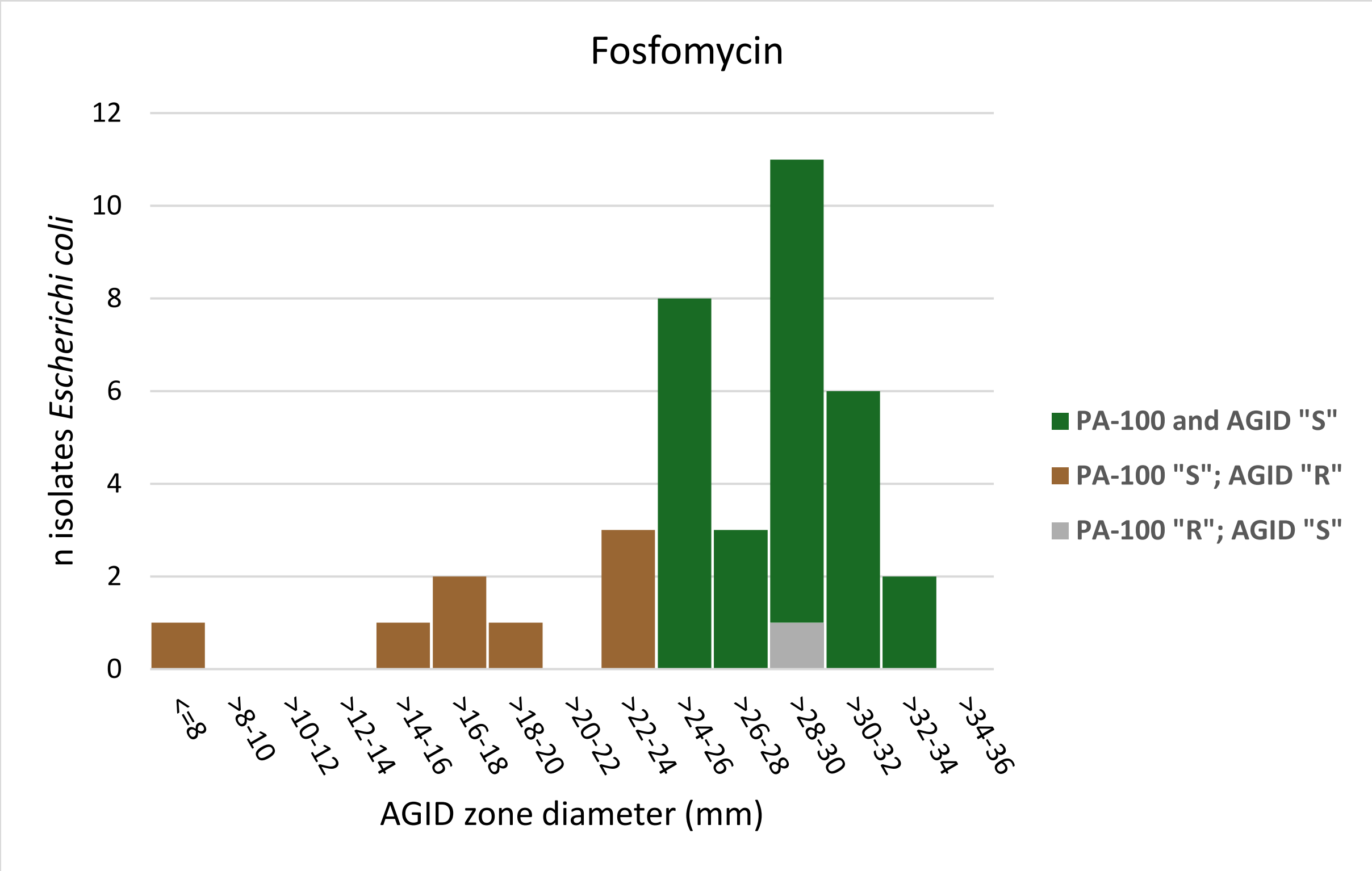

Supplement: Supplementary file 1 [file spectrum.00811-26-s0001.pdf]
